# Screening for distress in patients with primary brain tumor using distress thermometer: a systematic review and meta-analysis

**DOI:** 10.1186/s12885-018-3990-9

**Published:** 2018-02-02

**Authors:** Fangkun Liu, Jing Huang, Liyang Zhang, Fan Fan, Jindong Chen, Kun Xia, Zhixiong Liu

**Affiliations:** 1 Department of neurosurgery, Xiangya Hospital, Central South University (CSU), Changsha, China; 2 Department of Psychiatry, the Second Xiangya Hospital, Central South University, Changsha, Hunan 410011 China; 3 Chinese National Technology Institute on Mental Disorders, Hunan Key Laboratory of Psychiatry and Mental Health, Mental Health Institute of the Second Xiangya Hospital, Central South University, Chinese National Clinical Research Center on Mental Disorders (xiangya), Changsha, Hunan 410011 China; 4 The State Key Laboratory of Medical Genetics, School of Life Sciences, Central South University, Changsha, Hunan China

**Keywords:** Distress, Distress thermometer, Primary brain tumor, Glioblastoma, Meta-analysis

## Abstract

**Background:**

Patients with primary brain tumors are reported to have an elevated level of distress prevalence, due to the functional sequelae and the unfavorable prognosis, but the estimated prevalence of this disorder varies among studies. The Distress Thermometer (DT) is widely used distress screening tools to identify patients suffering from elevated psychosocial distress. The objective of this meta-analysis is to get a summarized estimate of distress prevalence in adult primary brain tumor patients screened by the DT instrument to identify distress in brain tumor patients.

**Method:**

We searched studies published in PubMed, PsycINFO, and Cochrane library through August 2017 and checked related reviews and meta-analyses for eligible studies. Studies were eligible if they were published in the peer-reviewed literature and evaluated distress level by Distress Thermometer. The prevalence of distress symptoms in patients with the intracranial tumor was estimated by study-level characteristics using stratified meta-analysis. The prevalence of distress level or symptoms during the follow-up examination at different time points was detected by secondary analysis of the longitudinal studies included.

**Results:**

Twelve studies including a total of 2145 brain tumor patients were included in this analysis. Eight used a cross-sectional design and four were longitudinal. The pooled prevalence of distress was 38.2% (95% confidence interval (CI) 28.7%–47.7%) for the overall sample. The pooled prevalence of distress DT ≥4 was 41.1% (642/1686, 95% CI 28.6%–53.5%) and the pooled prevalence of distress by DT ≥6 was 29.7% (137/459, 95% CI 19.5%–39.9%). The distress symptom did not decrease in follow-up studies (Relative Increase Ratio:1.02, 95% CI, (0.78, 1.35)). A huge heterogeneity in different studies was detected, and different screening scales were not compared.

**Conclusion:**

The high prevalence of distress becomes an enormous challenge for primary brain tumor patients. Routine screening and evaluation of distress in brain tumor patients may assist medical workers to develop proper interventions, which may lead to better quality of life and oncology management.

**Electronic supplementary material:**

The online version of this article (10.1186/s12885-018-3990-9) contains supplementary material, which is available to authorized users.

## Background

Distress is the emotional or mental discomfort under the circumstance of stressful life events [1–3]. Patients with distress suffer from a constellation of emotional and physical problems such as depression, insomnia, fatigue, pain, constipation and loss of concentration [2]. Brain tumor patients are reported to have an elevated level of distress prevalence, due to the severe functional sequelae and the unfavorable prognosis [4–6]. The high emotional distress experience results in significant emotional burden and greatly affected how patients cope with their diseases and their ability to follow treatment recommendations [7, 8]. These complications reduce health-related quality of life (HRQoL) and have a significant negative impact on prognosis as well as survival in brain tumor patients [5, 7]. A valid and practicable screening instrument for the diagnosis of distress in patients with intracranial tumor should be developed and studied. Different screening standards have been developed for the psychosocial diagnosis and support of cancer patients.

The National Comprehensive Cancer Network distress thermometer (NCCN-DT), a validated distress screening tool, has been widely used for the evaluation of psychiatric distress in cancer patients [2, 7], to improve the identification, management, and treatment of psychological distress [7]. The DT instrument uses a 0–10 scale to assess distress level from no distress to extreme distress [7]. A problem list is also included for patients to find the possible problems and concerns [5, 7]. Cancer patients are encouraged to use DT as part of their routine appointment preparation which makes them easier to talk to their doctors about the emotional effects caused by the diagnosis, symptoms, and treatment of cancer [9]. The Distress Thermometer has been employed in many studies and found to work well. Usually, patients scoring ≥4 are considered to have moderate distress symptoms which need intervention [2, 3, 9]. Also, some researchers recommended applying DT ≥ 6 for screening extreme distress in brain tumor patients [10, 11].

However, the estimated prevalence of this disorder varies among studies in primary neuro-oncological patients [5, 9, 11–13]. Different research design, sample size, research years and patients samples with different education level, marriage state, tumor grade and position contribute to the heterogeneity [5, 13, 14]. The purpose of the study is to obtain a reliable pooled distress prevalence in brain tumor patients measured by the DT and discuss the proper identification and treatment of this comorbidity of primary brain tumor.

## Methods

### Search strategy and inclusion criteria

**Fig. 1:**
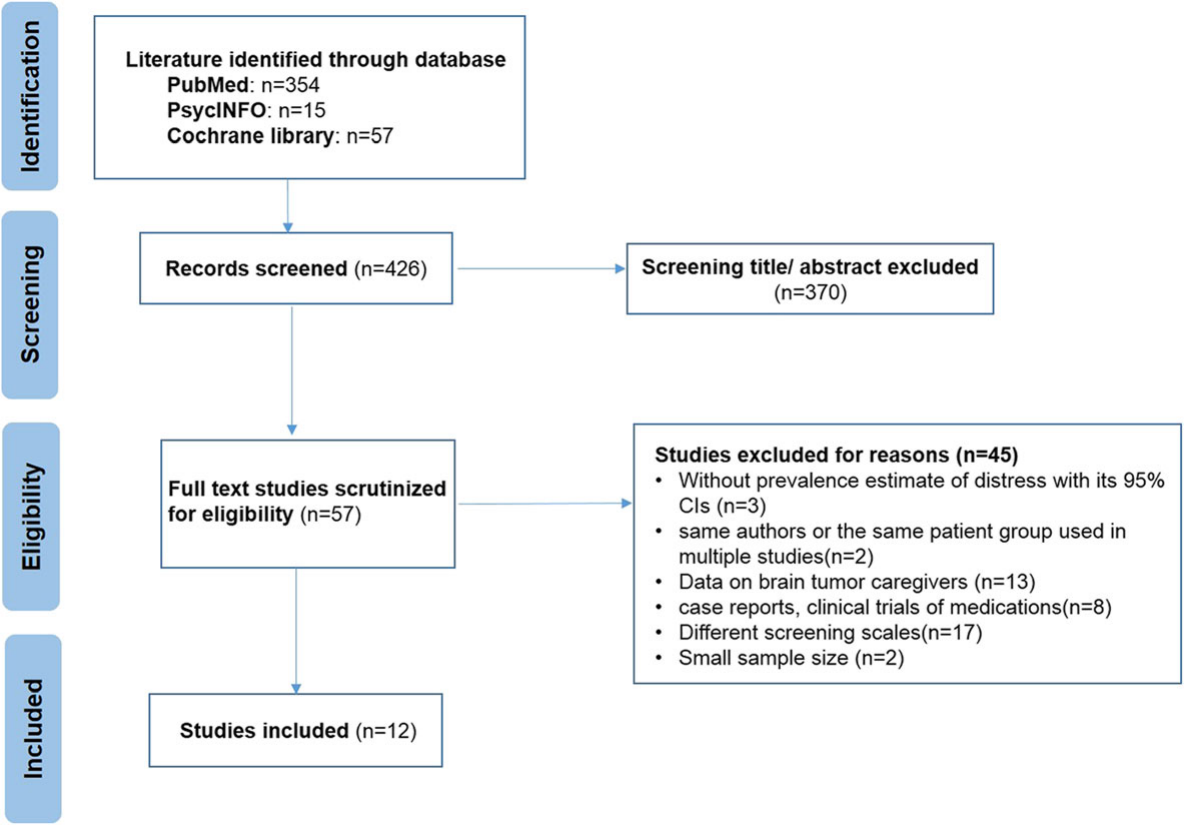
Meta-Analysis flowchart for identifying studies on the prevalence of distress among brain tumor patients

A literature search was performed using PubMed, PsycINFO, and Cochrane library with the following key words: “brain tumor” or “primary brain tumor” or “brain neoplasm” or “meningioma” or “glioblastoma” or “GBM” or “astrocytoma” or “oligodendroglioma” or “oligoastrocytoma” or “high-grade glioma” or “high-grade glioma” or “primary malignant brain tumor” or “intracranial tumors” or “neuro-oncological patients” and “distress” or “distress thermometer” or “psychiatric distress” or “distress symptom” or “emotional distress” or “mental distress”. We also searched reviews and meta-analyses to identify studies that may be missed in the former literature searches. Furthermore, all reference lists of the retrieved articles were obtained and reviewed in full text to search for additional eligible studies. Study authors were contacted to identify additional information if needed. PRISMA guidelines were used for this meta-analysis [15] (Fig. 1).

All studies met the following criteria were eligible for inclusion 1) used an observational or a randomized controlled trial before August 20, 2017; 2) provided distress prevalence in primary brain tumor patients with complication of distress ≥18 years old to ensure they can complete the questionnaire by themselves; 3) evaluated distress level by the National Comprehensive Cancer Network distress thermometer (NCCN-DT); 4) were published in peer-reviewed journals in English language; 5) For longitudinal studies, baseline pre-treatment data were included for the estimate of pooled prevalence of distress symptoms, and data at baseline and after 3 months were analyzed to study prevalence change over time.

Studies were excluded if: data from abstracts without full reports; studies included ≤30 patients; non–English-language studies; case reports. Studies were also excluded if it included tumors with cell origins that differed from that of the brain.

### Data extraction and quality assessment of included studies

Two investigators (FL and JH) independently extracted the following information from all eligible studies: study design, year, country or area, patients involved, tumor grade, education levels, DT cut-off, and prevalence. Table 1 summarized the included studies with the demographic and clinical characteristics. Publications potentially reporting data about distress were selected for full-text review and checked for eligibility. Any discrepancies were resolved by consensus, referring back to the original article. Three studies detected distress prevalence in the follow-up period were included and analyzed in our meta-analysis.

**Table 1:** Characteristics of studies included in the Meta-analysis

First author	Year	Country	Study design	Patients, n	Analytic Case, n (%)	Male patients, n (%)	Age, years, Mean ± SD (range)	WHO low-grade, n	WHO high-grade, n	Surgery,%	Married, %	White,%	When distress assessed initially	Distress cut-off
Rooney	2012	UK	longitudinal	154	133	57.4	54.2 ± 12.3 (19–76)	22	133	74.8	71	NA	during primary radiotherapy	DT ≥ 4/DT ≥ 6
Trad	2015	Australia	longitudinal	128	96	NA	NA	57	39	NA	NA	NA	newly diagnosis or first recurrence	DT ≥ 4
Goebel	2011	Germany	cross-sectional	159	159	48.4	55.2 ± 15.5(18–82)	87	72	NA	71.1	NA	Post-operation	DT ≥ 4/DT ≥ 6
Keir	2007	USA	cross-sectional	75	75	63	49 (24–70)	8	63	NA	NA	97	After diagnosis	DT ≥ 4/DT ≥ 6
Kvale	2009	USA	cross-sectional	50	50	58	53.3 ± 13.6 (20–85)	0	50	NA	NA	84	first to the Neuro-oncology clinic	DT ≥ 4
Renovanz	2013	Germany	longitudinal	134	134	35	52.7 ± 14.8 (18–81)	47	51	100	NA	NA	Pre-operation	DT ≥ 6
Rooney	2013	UK	longitudinal	154	154	60.9	55.9 ± 13.4	12	57	NA	NA	NA	shortly after starting chemo/radiotherapy	DT ≥ 4/DT ≥ 6
Goebel	2010	Germany	cross-sectional	150	150	43.3	53.2 ± 14.1 (18–79)	73	77	100	64.3	NA	1 week after/before operation	DT ≥ 6
Keir	2008	USA	cross-sectional	83	83	63.0	50 (25–70)	0	83	96.4	NA	96	After diagnosis	DT ≥ 4
Halkett	2015	Australia	cross-sectional	116	116	70.7	56 ± 13.3 (18–86)	0	116	NA	82.9	NA	Pre-chemoradiotherapy	DT ≥ 4
Randazzo	2017	USA	cross-sectional	829	798	54.0	51 (18–86)	218	576	NA	59	NA	Primary diagnosis or first recurrence	DT ≥ 4
Renovanz	2017	Germany	cross-sectional	244	173	53.2	51.0 ± 13.9 (21–78)	32	141	68	NA(72% with a partner)		first to the Neuro-oncology clinic	DT ≥ 6

*DT* Distress Thermometer, *NA* not applicable, *CI* confidence interval

### Statistical analysis

**Fig. 2:**
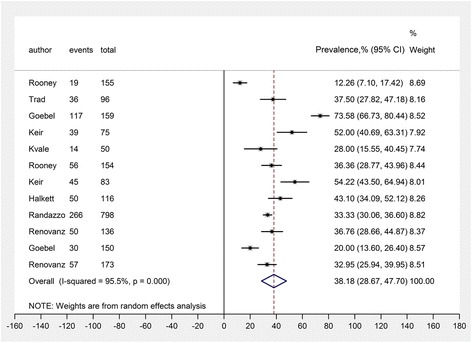
Forest plot for random-effects meta-analysis showing pooled prevalence of distress in the overall sample. CI, confidence interval

The statistical heterogeneity among studies was tested by Cochran’s Q statistic, *P* < 0.10 was considered of significance [16]. The quantity I^2^ that describes the percentage variation across studies that are attributed to heterogeneity was also assessed. An I^2^ ≥ 75% indicated significant heterogeneity. We used a random-effects model to calculate all point estimates of analyses and their 95% confidence interval (95% CI) (Fig. 2). Publication bias was evaluated using funnel plots and the Egger test. *P* < 0.10 was considered to represent statistically significant publication bias. The analysis was performed using Strata software (version 12.1; Stata Corp, College Station, TX). Forest plots were constructed as well. We also used stratified meta-analysis to compare results from different studies separately based on their characteristics (study design, country, sample size, year of the baseline survey, and cutoff score).

## Results

The overview of our search process was illustrated in Fig. 1. The initial search strategy identified 426 potentially articles: 354 from PubMed, 57 from Cochrane library, and 15 from PsycINFO. After screening the titles and abstracts according to the selection criteria, we excluded 370 studies. We also identified additional studies by reference scanning and previous meta-analysis or reviews. Overall, 12 eligible studies met the predetermined criteria for inclusion, including eight cross-sectional [4, 5, 9, 10, 13, 17–19] and four longitudinal studies [11, 12, 20, 21].

### Main associations of distress with brain tumor

These studies provided a total sample of 2145 brain tumor patients (median sample size = 179 patients, range = 50–798 patients). Four studies were conducted in the United States [4, 5, 9, 17], eight in other countries [10–13, 18–21]. These twelve studies were published between 2006 and 2015. Table 1 summarized the study characteristics and corresponding estimated prevalence with 95% CIs.

The pooled prevalence of distress was 38.2% (95% CI 28.7%–47.7%) in the overall sample with random-effects meta-analysis, ranging from 12.3% to 73.6% (Fig. 2). Significant evidence of between-study heterogeneity was observed between studies in the meta-analysis (I^2^ = 95.5%, *P* < 0.01). Studies with cut-off scores of ≥4 showed substantial distress 41.1% (642/1686, 95% CI 28.6%–53.5%) and studies with DT cut off score ≥ 6 showed substantial distress 29.7% (137/459, 95% CI 19.5%–39.9%).

The prevalence of distress symptoms by study-level characteristics using stratified meta-analysis was showed in Additional files 1 and 2. To examine consistency across different study designs with potential biases, we stratified data into subgroups on the basis of study design. There was significant difference between cross-sectional vs longitudinal studies (618/1604, 42.1% [95% CI, 29.9% to 54.2%] vs 161/541, 30.5% [95% CI, 15.9% to 45.0%]). A slightly lower prevalence of distress was detected in patients from USA than other countries (383/1161, 35.5% [95% CI, 21.4% to 49.6%] vs 396/984, 40.0% [95% CI, 26.3% to 53.8%]), *p* < 0.01. Significant differences in prevalence estimates were also noted when studies were stratified by year ≥2010 vs year < 2010 (681/1937, 36.1% [95% CI, 25.1%–47.1%] vs 98/208, 45.0% [95% CI, 29.2%–70.8%]). We then detected the prevalence difference between large sample size (sample ≥ 100) vs small sample size (sample < 100) (645/1841, 35.9% [95% CI, 23.9% to 48.0%] vs 134/304, 43.1% [95% CI, 31.5%–54.1%]). (Additional files 1 and 2). No further analysis was performed for comparison of different position or type of brain tumor. Among all the subgroups we detected, heterogeneity was in part explained by survey country (*P* < 0.01), sample size (*P* < 0.01), distress scale (*P* < 0.01) and study design (*P* < 0.01).

There were 3 longitudinal studies provided results on the prevalence of distress during further analysis [12, 20, 21]. The prevalence of distress level did not decrease over time (Relative Increase Ratio:1.02, [95% CI, (0.78, 1.35)]) (Table 2).

**Table 2:** Secondary analysis of three longitudinal studies reporting distress prevalence in the follow-up period

							Baseline			Follow-up			Comparison
First author	Year	Country	Study design	When distress assessed initially	Distress cut-off	Follow-up	No. of patients with Distress, n	Total number of Patients assessed, n	Prevalence of Distress,%(95% Cl)	No. of patients with Distress, n	Total number of Patients assessed, n	Prevalence of Distress,%(95% Cl)	Relative Increase Ratio,%(95% Cl)
Rooney	2012	UK	longitudinal	during primary radiotherapy	DT ≥ 4	3 mo	19	155	12.3 (7.1–17.4)	15	108	13.9 (7.4–20.4)	0.81 (0.42,1.54)
Trad	2015	Australia	longitudinal	newly diagnosis or first recurrence	DT ≥ 4	3 mo	36	96	37.5 (27.8–47.2)	9	12	75.0 (50.5–99.5)	1.57 (0.89,2.77)
Rooney	2013	UK	longitudinal	shortly after starting chemo/radiotherapy	DT ≥ 4	3 mo	56	154	36.4 (28.8–44.0)	37	103	35.9 (26.7–45.2)	0.99 (0.78,1.35)

*DT* Distress Thermometer, *CI* confidence interval

### Publication bias

**Fig. 3:**
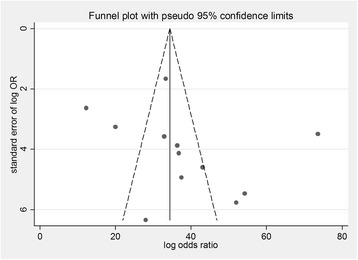
Funnel plot for the included studies that examined small study effects

Publication bias was investigated by funnel plot (Fig. 3) and Egger test. There was no evidence of small studies effect (Egger test *P* = 0.32).

## Discussion

This study provides strong clinical evidence showing primary brain tumor patients have a high level of distress prevalence from 12 observational studies. Based on our findings, patients with intracranial tumor have a higher prevalence of distress compared with a non-clinical population, which ranges between 5% to 27% [22–26]. The high risk of emotional complications and their harms in brain tumor patients become an enormous challenge for disease management. The distress prevalence in patients with intracranial tumor is not higher than that in patients with lung cancer (61.6%) [27] or bone marrow transplant patients (43.0%) [28]. The possible reasons could be the quick disease progression of a malignant brain tumor or early interventions by some of the clinical practitioners [17, 29–31]. Fatigue, pain, anxiety, and depression are among the most troubling symptom associated with the prevalence of distress in brain tumor patients which will result in a poorer overall survival and decreased health-related quality of life (HRQoL) [17, 32]. Caregivers also have severe distress experience according to some studies [9, 10]. The mental and physical distress would lead to low quality of life, predicate poor therapeutic effect, and satisfaction with health care [13]. Routine screening and evaluation of distress in brain tumor patients may assist medical workers to develop proper intervention [13, 33, 34], which may improve prognosis [35]. More studies should be planned to identify the risk factors of brain tumor patients and integrate appropriate interventions to improve HRQoL.

The study has some limitations. A huge heterogeneity in different studies was detected. After sub-group analysis, we found that different study design, sample size, study country, cut-off point and year published contributed to the heterogeneity. The effect of tumor size and grade on distress remains controversial [13, 36]. Tumor biology has an influence on cognition function and physiological environment in patients [37, 38], and intracranial tumors could invade and affect function area, but they did not alter the Distress Thermometer scoring according to Goebel’s research [13]. However, similar studies using Hospital Anxiety and Depression Scale (HADS) and Beck Depression Inventory (BDI) to assess distress, anxiety and depressive symptoms, have found that patients with meningioma are more likely to develop emotional stress, but other studies did not support this finding [36, 39–42].

Our findings are based on one single screening method, the Distress Thermometer produced by the National Comprehensive Cancer Network (NCCN) [2]. There are other screening scales used in clinical setting to assess the distress-related symptoms such as depression, anxiety, and fatigue [8, 43–50]. For example, the hospital anxiety and depression scale (HADS) was used to identify distress in some studies by which DT was compared [47]. Simone Goebel et al. found that the ability of DT to screening distress in brain tumor patients was efficient and excellent by comparing different DT scores with HADS [13]. And correlation analysis for the relationship between DT scores and HADS anxiety and depression found that they are closely relevant. Distress levels could reflect emotional problems including anxiety and depression. To date, there seems no consensus to define the best-standardized scale to evaluate distress-related symptoms in clinical settings. In the future studies, different scales should be compared to analyze their accuracy and consistency in the identification of disease.

It would be better if we can monitor the distress prevalence change during a routine follow-up examination [35]. There were limited studies monitoring distress change over time and recording relative indicators during this period [12, 20, 21]. We hope more studies will track distress over time in combination with feedback information to provide better insight into this field and develop appropriate supportive care options. And study design including healthy control group or extracranial tumor patients is recommended.

## Conclusions

The high prevalence of distress becomes an enormous challenge for primary brain tumor patients. The role of distress in intracranial tumor patients should be studied and understood to develop proper management and maintain the good Health-related quality of life. More studies to track distress over time are needed to develop appropriate supportive care options for intracranial tumor patients.

## Additional files

Additional file 1: Meta-analysis of the prevalence of distress symptoms among brain tumor patients stratified by study-level characteristics. (DOC 40 kb)

Additional file 2: Meta-analysis of the prevalence of distress symptoms among brain tumor patients stratified by study design (A), country (B), sample size (C), year (D) and distress scale cut-off (E). CI, confidence interval. (ZIP 6618 kb)

## Abbreviations

BDI: Beck Depression Inventory
DT: The Distress thermometer
HADS-D: Depression Subscale of Hospital Anxiety and Depression Scale
HRQoL: Health-related quality of life
NA: not applicable
