# Supplementary material for: Screening for distress in patients with primary brain tumor using distress thermometer: a systematic review and meta-analysis
Source: BMC Cancer. 2018 Feb 2;18:124. doi: 10.1186/s12885-018-3990-9 (PMC5797347; doi:10.1186/s12885-018-3990-9)
Supplement: Supplementary file 1 — Meta-analysis of the prevalence of distress symptoms among brain tumor patients stratified by study-level characteristics. (DOC 40 kb) [file 12885_2018_3990_MOESM1_ESM.doc]

**Supplement 1.** Meta-analyses of the prevalence of distress symptoms among brain tumor patients stratified by study-level characteristics.

|  | No. of studies, n | No. of patients with Distress, n | Total number of Patients, n | Prevalence of Distress,%(95%Cl) | P value for subgroup differences |
| --- | --- | --- | --- | --- | --- |
| Study Design |  |  |  |  |  |
| Longitudinal | 4 | 161 | 541 | 30.5(15.9-45.0) | < 0.01 |
| Cross-sectional | 8 | 618 | 1604 | 42.1(29.9-54.2) |  |
| Country |  |  |  |  |  |
| USA | 5 | 383 | 1161 | 35.5(21.4-49.6) | < 0.01 |
| Others | 7 | 396 | 984 | 40.0(26.3-53.8) |  |
| Sample size |  |  |  |  |  |
| ≥100 | 8 | 645 | 1841 | 35.9(23.9-48.0) | < 0.01 |
| <100 | 4 | 134 | 304 | 43.1(31.5-54.7) |  |
| Year |  |  |  |  |  |
| ≥2010 | 9 | 681 | 1937 | 36.1(25.1-47.1) | < 0.01 |
| <2010 | 3 | 98 | 208 | 45.0(29.2-70.8) |  |
| Distress scale Cut-off |  |  |  |  |  |
| DT≥4 | 9 | 642 | 1686 | 41.1(28.6-53.5) | < 0.01 |
| DT≥6 | 3 | 137 | 459 | 29.7(19.5-39.9) |  |

DT, distress thermometer; CI, confidence interval.
