# Supplementary figures and images for: Screening for distress in patients with primary brain tumor using distress thermometer: a systematic review and meta-analysis
Source: BMC Cancer. 2018 Feb 2;18:124. doi: 10.1186/s12885-018-3990-9 (PMC5797347; doi:10.1186/s12885-018-3990-9)

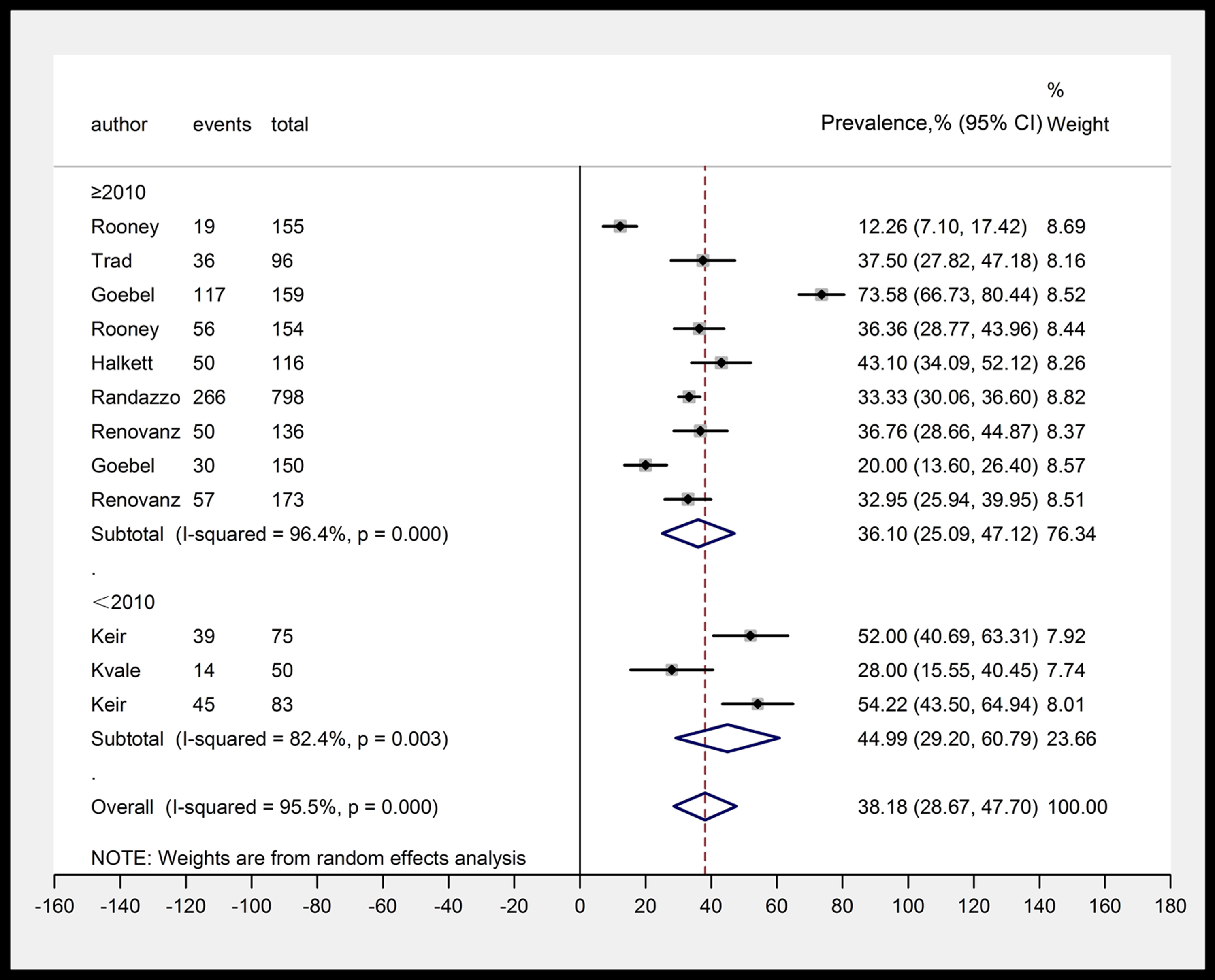

Supplement: Supplementary file 2 — Meta-analysis of the prevalence of distress symptoms among brain tumor patients stratified by study design (A), country (B), sample size (C), year (D) and distress scale cut-off (E). CI, confidence interval. (ZIP 6618 kb) [file 12885_2018_3990_MOESM2_ESM.zip › Supplement 2DR2.tif]

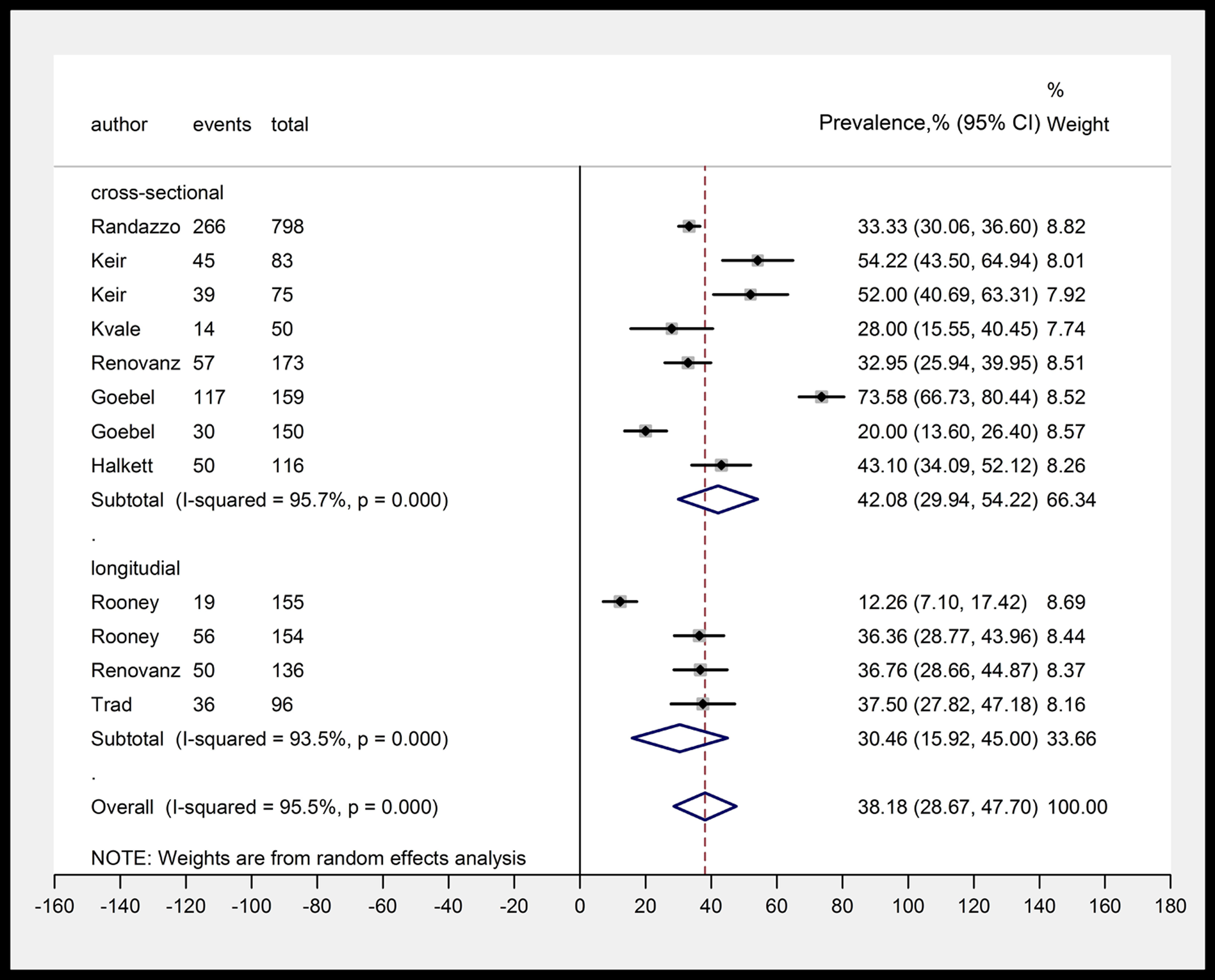

Supplement: Supplementary file 2 — Meta-analysis of the prevalence of distress symptoms among brain tumor patients stratified by study design (A), country (B), sample size (C), year (D) and distress scale cut-off (E). CI, confidence interval. (ZIP 6618 kb) [file 12885_2018_3990_MOESM2_ESM.zip › Supplement 2AR2.tif]

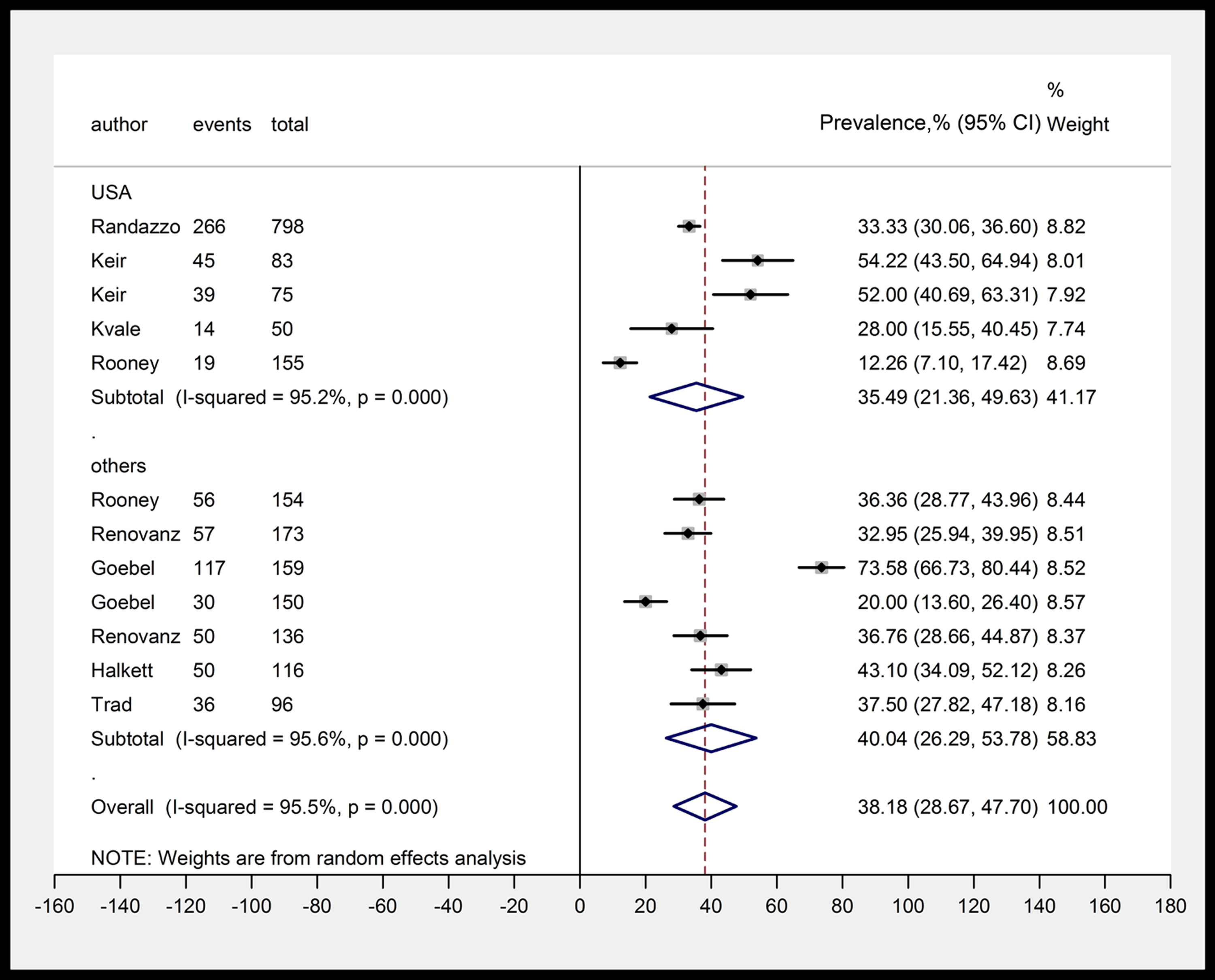

Supplement: Supplementary file 2 — Meta-analysis of the prevalence of distress symptoms among brain tumor patients stratified by study design (A), country (B), sample size (C), year (D) and distress scale cut-off (E). CI, confidence interval. (ZIP 6618 kb) [file 12885_2018_3990_MOESM2_ESM.zip › Supplement 2BR2.tif]

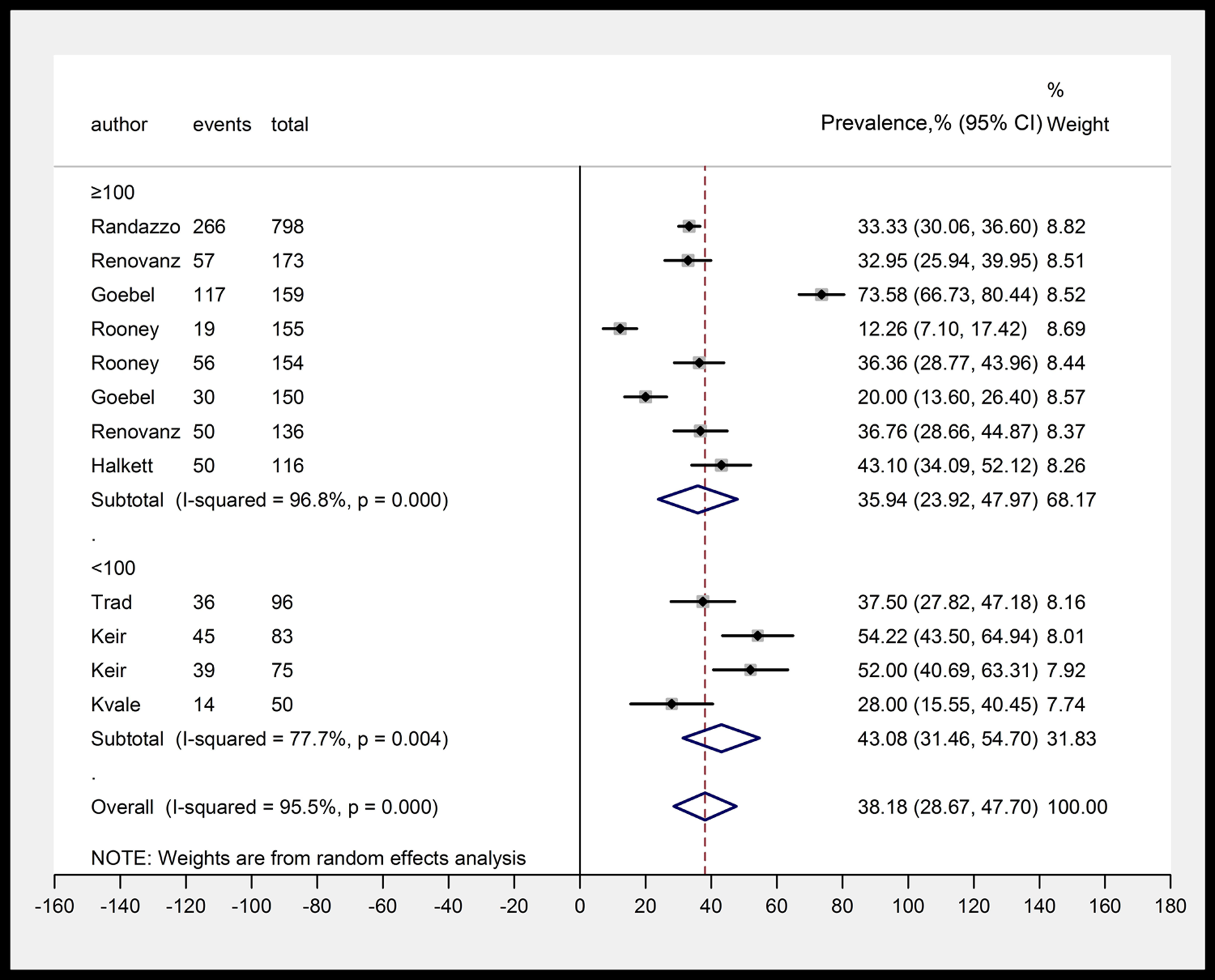

Supplement: Supplementary file 2 — Meta-analysis of the prevalence of distress symptoms among brain tumor patients stratified by study design (A), country (B), sample size (C), year (D) and distress scale cut-off (E). CI, confidence interval. (ZIP 6618 kb) [file 12885_2018_3990_MOESM2_ESM.zip › Supplement 2CR2.tif]

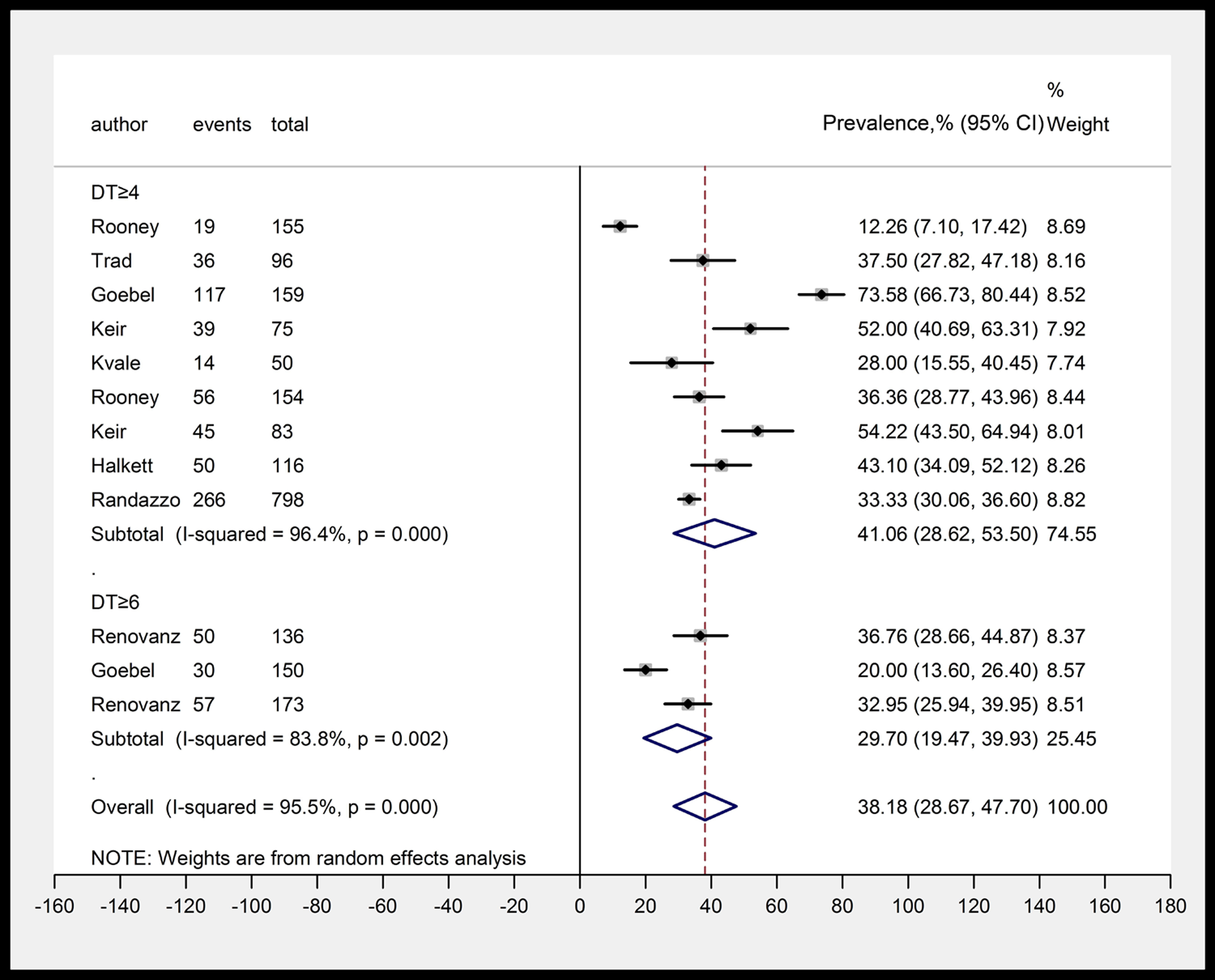

Supplement: Supplementary file 2 — Meta-analysis of the prevalence of distress symptoms among brain tumor patients stratified by study design (A), country (B), sample size (C), year (D) and distress scale cut-off (E). CI, confidence interval. (ZIP 6618 kb) [file 12885_2018_3990_MOESM2_ESM.zip › Supplement 2ER2.tif]
